# Feasibility of anomaly score detected with deep learning in irradiated breast cancer patients with reconstruction

**DOI:** 10.1038/s41746-022-00671-0

**Published:** 2022-08-23

**Authors:** Dong-Yun Kim, Soo Jin Lee, Eun-Kyu Kim, Eunyoung Kang, Chan Yeong Heo, Jae Hoon Jeong, Yujin Myung, In Ah Kim, Bum-Sup Jang

**Affiliations:** 1 Department of Radiation Oncology, Seoul National University Hospital, Seoul, Korea; 2 College of Medicine, Seoul National University, Seoul, Korea; 3 Department of Surgery, Seoul National University Bundang Hospital, Seoul National University College of Medicine, Seongnam, Korea; 4 Department of Plastic and Reconstructive Surgery, Seoul National University Bundang Hospital, Seongnam, Korea; 5 Department of Radiation Oncology, Seoul National University Bundang Hospital, Seongnam, Korea

**Keywords:** Outcomes research, Biotechnology

## Abstract

The aim of this study is to evaluate cosmetic outcomes of the reconstructed breast in breast cancer patients, using anomaly score (AS) detected by generative adversarial network (GAN) deep learning algorithm. A total of 251 normal breast images from patients who underwent breast-conserving surgery were used for training anomaly GAN network. GAN-based anomaly detection was used to calculate abnormalities as an AS, followed by standardization by using z-score. Then, we reviewed 61 breast cancer patients who underwent mastectomy followed by reconstruction with autologous tissue or tissue expander. All patients were treated with adjuvant radiation therapy (RT) after reconstruction and computed tomography (CT) was performed at three-time points with a regular follow-up; before RT (Pre-RT), one year after RT (Post-1Y), and two years after RT (Post-2Y). Compared to Pre-RT, Post-1Y and Post-2Y demonstrated higher AS, indicating more abnormal cosmetic outcomes (Pre-RT vs. Post-1Y, *P* = 0.015 and Pre-RT vs. Post-2Y, *P* = 0.011). Pre-RT AS was higher in patients having major breast complications (*P* = 0.016). Patients with autologous reconstruction showed lower AS than those with tissue expander both at Pre-RT (2.00 vs. 4.19, *P* = 0.008) and Post-2Y (2.89 vs. 5.00, *P* = 0.010). Linear mixed effect model revealed that days after baseline were associated with increased AS (*P* = 0.007). Also, tissue expander was associated with steeper rise of AS, compared to autologous tissue (*P* = 0.015). Fractionation regimen was not associated with the change of AS (*P* = 0.389). AS detected by deep learning might be feasible in predicting cosmetic outcomes of RT-treated patients with breast reconstruction. AS should be validated in prospective studies.

## Introduction

Breast reconstruction after mastectomy has been more widely used in breast cancer patients^1^. Breast reconstruction has the advantage of providing physical and psychological relief to patients who underwent breast cancer surgery^2^. Aligned with the prolonged life expectancy due to the early detection and treatment advances in breast cancer^3,4^, cosmetic satisfaction achieved by breast reconstruction after mastectomy is important for patients. Particularly, most breast cancer patients require radiation therapy (RT) after surgery, and RT is known to cause capsular contracture or deformity of the reconstructed breast. Therefore, cosmetic evaluation of breast reconstruction patients who underwent RT is more necessary. Since satisfactory cosmetic outcomes after breast cancer surgery can lead to a better quality of life (QOL), physicians are concerned about improving cosmetic results as well as clinical outcomes.

To evaluate cosmetic outcomes, there are a few established criteria. The Breast-Q questionnaire is a validated tool for measuring health-related QOL and satisfaction in patients with breast reconstruction^5–7^, albeit measurement is based on a subjective evaluation. The medical photographs taken according to standardized guidelines can be used for evaluation^8^, but breast photos cannot be an objective indicator due to the potential judgement bias by clinicians. To gain objectivity in cosmetic evaluation, several methods have been suggested, including breast retraction assessment (BRA) and Breast Cancer Conservative Treatment cosmetic results (BCCT.core) software^9,10^. BRA measures the distances between sternal notch-nipples and nipples-breast outline, which does not reflect skin alteration or scar problems^11^. The BCCT.core program automatically evaluates medical photographs of the patient and has been validated in several studies^10,12,13^. The BCCT.core software is designed for evaluating four categories of cosmesis: excellent, good, fair, and poor^12^. However, medical photography taking naked upper body might cause uncomfortable feelings for patients. Further, the BCCT.core software has limitation with regard to the lack of 3-dimentional (3D) volume information^14^.

Recently, deep learning methods have been applied in medical areas for anomaly detection based on training normal images^15^. Generative adversarial network (GAN) is a type of neural computational network model for two networks training simultaneously^16^. The final GAN-based anomaly model could capture abnormal features from new images based on the trained normal images^17^, and several studies validated its feasibility^18,19^. Compared to BCCT.core software, the GAN-based approach for detecting anomalies from computed tomography (CT) images did not cause additional discomfort for patients. In addition, continuous and numerical measurement of AS could make it possible for patients or clinicians to evaluate cosmetic outcomes given that AS is defined as the sum of loss of images and loss of features between normal images and reconstructed images.

Thus, the purpose of the current study is to develop GAN-based model that can generate AS for assessment of cosmetic results from mastectomy patients who underwent immediate reconstruction and to investigate its implication with regard to clinical factors.

## Results

### Patient characteristics

We retrospectively reviewed 61 breast cancer patients who underwent mastectomy followed by immediate reconstruction and adjuvant RT. Among the 61 patients, 39 (64.0%) received total mastectomy (TM)/radical mastectomy, 16 (26.2%) did nipple-sparing mastectomy (NSM), and 6 (9.8%) did skin-sparing mastectomy (SSM). As for the type of reconstruction, 47 used autologous tissue and 14 had tissue expander. Of all, 53 patients (86.9%) received conventional fractionated RT, and 8 (13.1%) received hypofractionated RT. Majority of patients received no neoadjuvant chemotherapy (*N* = 43, 70.5%), meanwhile, substantial patients received adjuvant chemotherapy (*N* = 39, 63.9%). Radiotherapy was delivered by using 3-dimensional (3D) conformal (*N* = 48, 78.7%) or intensity modulated radiation therapy (IMRT) technique (*N* = 13, *N* = 21.3%). Patients with Body mass index (BMI) ≤ 23 and those with BMI > 23 were distributed well. Time interval between pre-RT CT and Post-1Y CT was 417 days [interquartile range (IQR), 343–512 days], and interval between pre-RT CT and Post-2Y CT was 803 days (IQR, 741–951 days). AS of Pre-RT, Post-1Y and Post-2Y were 1.99 (range, −0.65 to 19.40), 2.92 (range, −0.16 to 11.50) and 2.94 (range, −0.36 to 14.35), respectively. Detailed characteristics of the study population are summarized in Table 1.

**Table 1:** Patient Characteristics of target dataset (*N* = 61).

Variables		*N* (%)
Age (Year)	≤45	32 (52.5%)
	>45	29 (47.5%)
Major Complication	No	51 (83.6%)
	Yes	10 (16.4%)
Mastectomy	Total/radical	39 (64.0%)
	Nipple-sparing	16 (26.2%)
	Skin-sparing	6 (9.8%)
Type of reconstruction	Autologous	47 (77.0%)
	Tissue Expander	14 (23.0%)
RT fractionation	Conventional	53 (86.9%)
	Hypofractionated	8 (13.1%)
Neoadjuvant chemotherapy	No	43 (70.5%)
	Yes	18 (29.5%)
Adjuvant chemotherapy	No	21 (34.4%)
	Yes	39 (63.9%)
	Missing	1 (1.6%)
RT plan	3D	48 (78.7%)
	IMRT	13 (21.3%)
Tumor bed boost	No	49 (80.3%)
	Yes	12 (19.7%)
The use of bolus	No	59 (96.7%)
	Yes	2 (3.3%)
Body mass index (kg/m^2^)	≤23	32 (52.5%)
	>23	29 (47.5%)
Pre-RT CT to Post-1Y CT (days)		417 (IQR 343–512)
Pre-RT CT to Post-2Y CT (days)		803 (IQR 741–951)
Pre-RT Anomaly score		1.99 (range, −0.65 to 19.40)
Post-1Y Anomaly score		2.92 (range, −0.16 to 11.50)
Post-2Y Anomaly score		2.94 (range, −0.36 to 14.35)

*RT* Radiation therapy, *3D* 3-dimensional, *IMRT* Intensity modulated radiation therapy, *CT* Computed tomography, *1Y* 1-year, *2Y* 2-year, *IQR* Interquartile range.

### Distribution of anomaly score

**Fig. 1:**
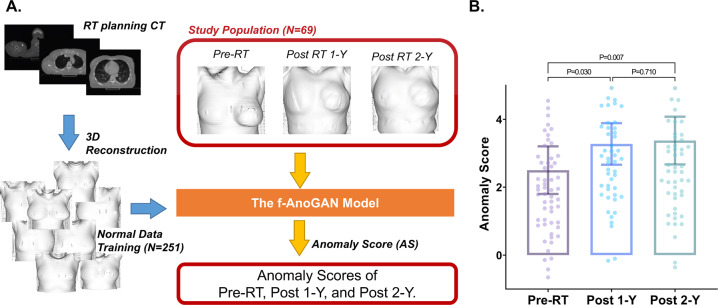
Overview of generation of AS using developed f-AnoGAN model and pairwise comparison of AS. **A** Graphical representation of process for developing the f-AnoGAN model with normal image data from RT planning CT and generation of anomaly score by developed model in time-series manner. **B** Pairwise comparison of anomaly score among pre-RT, post 1-Y, and post 2-Y time points. P-value was estimated by pairwise paired T-test. The 95% confidence intervals are drawn as error bars at each point. 3D 3-dimensional, f-AnoGAN Fast anomaly generative adversarial network, RT Radiation therapy, CT Computed tomography, 1-Y 1 year, 2-Y 2 years.

The f-AnoGAN model was trained with 3D-reconstructed images from RT planning CT images in patients receiving breast conserving therapy. Then, 3D-reconstructed from CT images in patients who received mastectomy and immediate reconstruction were collected in a time-series manner at Pre-RT, Post-1Y and Post-2Y time points. The developed f-AnoGAN model generated AS. Graphical representation of this process is depicted in Fig. 1A. Afterward, we compared the distribution of AS among time points (Fig. 1B). We found that AS of Pre-RT was significantly different compared to Post-1Y (*P* = 0.015) and Post-2Y (*P* = 0.011). There was no significant difference in AS between Post-1Y and Post-2Y (*P* = 0.980).

**Fig. 2:**
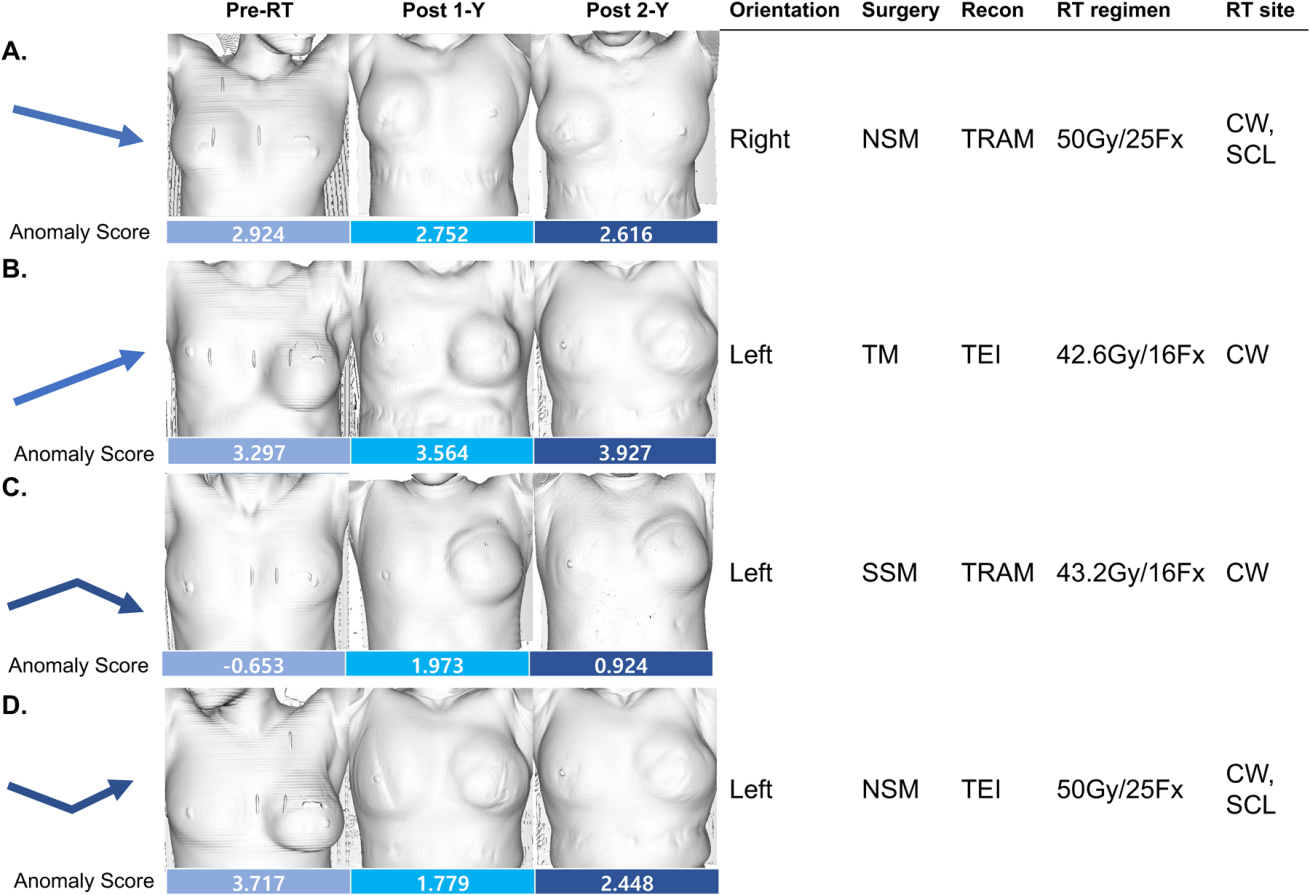
The four patterns of change in anomaly score (AS) in terms of radiation therapy, according to the type of breast cancer surgery and reconstruction method. **A** The decreasing trend of AS in patients who received right NSM with TRAM flap reconstruction. **B** The increasing trend of AS in patients who underwent left total mastectomy followed by TEI. **C** The increasing-decreasing trend of AS in patients who received left SSM with TRAM flap reconstruction. **D** The decreasing-increasing trend of AS in patients who underwent NSM followed by TEI. RT radiation therapy, 1-Y 1 year, 2-Y 2 years, NSM nipple-sparing mastectomy, TEI tissue-expander insertion, TRAM transverse rectus abdominis muscle, Fx fractions, CW chest wall, SCL subclavian lymph node, SSM skin-sparing mastectomy.

**Fig. 3:**
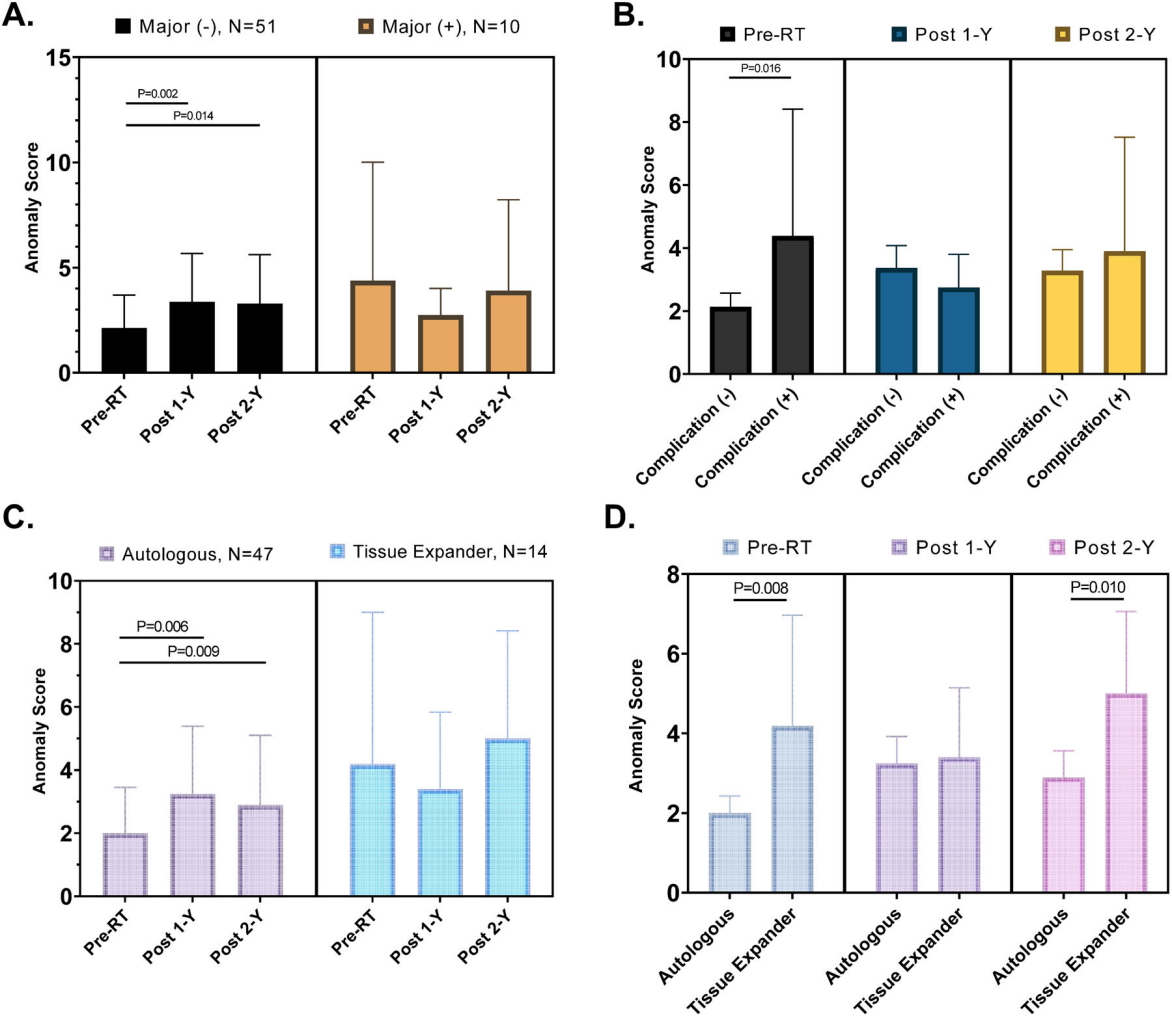
Comparative analyses using anomaly score. **A** Comparison of anomaly score among Pre-RT, Post-1Y, and Post-2Y time points, stratified by major complication event. **B** In each time point, anomaly score is compared according to complication event. **C** Stratified by reconstruction type, anomaly scores according to three time points are compared. **D** In each time point, anomaly score from patients with autologous tissue and tissue expander is compared. *P*-value was estimated by pairwise paired *T*-test. *P*-value was estimated by pairwise paired T-test. The 95% confidence intervals are drawn as error bars at each point. RT Radiation therapy, 1-Y 1 year, 2-Y 2 years.

At the patient level, we classified the four patterns of change in AS. The decreasing trend of AS was shown in patients who received NSM with immediate transverse rectus abdominis muscle (TRAM) flap reconstruction (Fig. 2A), implying better cosmetic outcome. The increasing trend of AS was found in patients who received TM and immediate reconstruction with tissue expander insertion (TEI) (Fig. 2B). We also found that the increasing-decreasing (Fig. 2C) and decreasing-increasing (Fig. 3D) trend of AS in patients with SSM with TRAM and NSM with TEI, respectively. Overall, NSM/SSM and TRAM seem to be related with lower anomaly score than TM and TEI.

With a median follow-up of 28.3 months, we found 14 major complication events (16.4%) in the study population. Patients who experienced major complication demonstrated no significant AS changes among three time points (Fig. 3A), however, they showed significantly higher AS than those without major complication at pre-RT (*P* = 0.016, Fig. 3B). We observed that there was differential time effect in patients with autologous reconstruction after mastectomy. Compared with Pre-RT AS, Post-1Y and Post-2Y AS were significantly elevated in autologous-reconstructed patients (Fig. 3C). However, there was no specific trend of AS in patients with TEI. TEI showed significantly higher AS than autologous reconstruction at Pre-RT (*P* = 0.008) and Post-2Y (*P* = 0.010) (Fig. 3D). However, there are no difference in AS between TEI and autologous reconstruction at Post-1Y.

### Longitudinal analysis

We used a multivariable LME model to analyze time-effect on AS considering potential confounding factors. The results are listed in Table 2. Multivariate LME analysis revealed that day after RT (*β* = 0.004, *P* = 0.007) and TEI (*β* = 2.223, *P* = 0.015) were significant factors to be associated with AS. ﻿Meanwhile, LME model showed no statistically significant interaction effect of reconstruction type (*P* = 0.563) and RT fractionation (*P* = 0.389) over time. We found that age had marginally positive correlation with AS (*β* = 0.073, *P* = 0.078), but other variables including mastectomy type (*P* = 0.627), RT to SCN (*P* = 0.154), RT to IMN (*P* = 0.840), boost RT (*P* = 0.295), RT plan (*P* = 0.865), major complication (*P* = 0.930), and BMI (*P* = 0.364) have no significant impact on the change of AS.

**Table 2:** Linear mixed model predicting anomaly score.

Variables	Coefficient	95% CI	*P*-value
Days after RT	0.004		0.007
Type of reconstruction			
Autologous	0.000	(base)	
Tissue expander	2.223	(0.431–4.016)	0.015
Type x Days after baseline	0.000	(−0.001–0.002)	0.563
RT fractionation			
Conventional	0.000	(base)	
Hypofractionated	0.485	(−2.149 to 3.120)	0.718
Fractionation x Days after RT	−0.001	(−0.004 to 0.002)	0.389
Mastectomy			
Total/Radical	0.000	(base)	
NSM/SSM	−0.309	(−1.556 to 0.938)	0.627
RT to SCL			
No	0.000	(base)	
Yes	1.094	(−0.412 to 2.600)	0.154
RT to IMN			
No	0.000	(base)	
Yes	−0.229	(−2.460 to 2.002)	0.840
Boost			
No	0.000	(base)	
Yes	0.909	(−0.792 to 2.610)	0.295
RT plan			
3D	0.000	(base)	
IMRT	−0.186	(−2.338 to 1.966)	0.865
Major complication			
No	0.000	(base)	
Yes	0.077	(−1.647 to 1.802)	0.930
Age (year)	0.073	(−0.008 to 0.153)	0.078
BMI (kg/m^2^)			
≤23	0.000	(base)	
>23	0.570	(−0.660 to 1.800)	0.364
Intercept	−2.390	(−6.701 to 1.920)	0.277

*P*-value by a linear mixed-effect model.

*CI* Confidence interval, *NSM* Nipple-sparing mastectomy, *SSM* Skin-sparing mastectomy, *SCL* Subclavian lymph node, *IMN* Internal mammary node, *3D* 3-dimensional, *IMRT* Intensity modulated radiation therapy, *BMI* Body mass index.

**Fig. 4:**
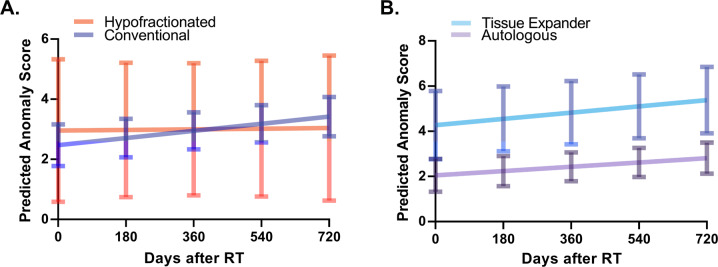
Change of AS according to RT fractionation and reconstruction type in time-dependent manner. Comparison of predicted anomaly score generated by linear mixed effect model between patients receiving hypofractionated and those receiving conventional fractionation of RT (**A**) and between patients reconstructed with tissue expander and those with autologous tissue (**B**). The 95% confidence intervals are drawn as error bars at each point. RT Radiation therapy.

Based on established LME model, we predicted the change of AS according to RT fractionation and reconstruction type in time-dependent manner. As shown in Fig. 4A, there was no significant difference in change of AS between patients who received hypofractionated and those who treated with conventional fractionated RT across all time points (*P* = 0.389). In a meanwhile, we observed significant difference in change of AS in all time points between TEI and autologous reconstruction (Fig. 4B). The gap widened over time, suggesting a consistently better cosmesis of autologous reconstruction compared with TEI after RT: Contrast = 2.2 (*P* = 0.015), 2.3 (*P* = 0.008), 2.4 (*P* = 0.005), 2.5 (*P* = 0.004), and 2.6 (*P* = 0.004) at 0, 180, 360, 540, and 720 days after RT, respectively.

## Discussion

The aesthetic results with respect to treatment of breast cancer is important for patient’s quality of life. This led to the advancement of techniques for breast-conserving surgery and oncoplastic breast surgery. Further, reconstruction after mastectomy in breast cancer patients is increasing. According to the registry of Korean Breast Cancer Society, the number of patients who underwent breast reconstruction surgery between 2002 and 2013 tripled^1^. Cosmetic evaluation for breast can be largely divided into subjective and objective considerations. As for the subjective assessment, BREAST-Q questionnaire is designed to measure the patient’s satisfaction with breast and psychosocial/physical well-being^6,7^. A prospective multicenter cohort study by Jagsi et al. utilized BREAST-Q questionnaire to evaluate patient-reported satisfaction in patients who received postmastectomy RT (PMRT) and reconstruction surgery. Also, there is the modified Garbay scale to assess the aesthetic results by rating 5 subscales: volume, shape, placement of breast, inframammary fold, and scars. However, limitation exists in that inter-rater agreement is low, even when performed by experts^20^. As an objective indicator for physicians, BCCT.core software using medical 2D photographs has been largely used due to the its reproducibility and reliability in terms of aesthetic assessment^10–13^. As mentioned earlier, it may cause psychological discomfort to patients when medical photographs are taken with their tops off. Moreover, it only uses a frontal view of photography, which entailed lack of volume information for processing the software. In a mean time, recently, anomaly detection using deep learning has evolved in oncology area, particularly screening and detection of cancer. Several studies facilitated GAN-based anomaly detection to diagnoses anomalous lesions in ultrasound images of breast^16^ and digital breast tomosynthesis^17^. Myung et al.^21^ newly published machine learning approaches for predicting complication in reconstructed breast cancer patients, though it did not provide cosmetic evaluation. To our knowledge, no research has been found to evaluate cometic outcome using by the GAN-based approach and its association with major complication after breast reconstruction and PMRT. Our study provides a novel information of GAN-based cosmetic evaluation for patients with breast reconstruction.

The present study analyzed 61 breast cancer patients who underwent immediate reconstruction with PMRT using f-AnoGAN algorithm. We generated AS that can detect abnormalities of reconstructed breast and quantify them as a continuous numerical measurement. Moreover, we used CT images achieved in regular follow-up imaging, which indicates no possibility of additional discomfort for patients. We found pre-RT AS was significantly higher in patients with major complications, in a mean time, reconstruction with autologous tissue showed lower AS than TEI. Also, we observed the day after RT and TEI were significant factors to predict AS, while RT fractionation showed no interaction on AS. LME model also revealed that autologous reconstruction had better cosmetic outcomes after RT in all time points, while hypofractionation and conventional fractionation showed no significant difference in change of AS. These results were consistent with other existing research results. Ho et al. reported TEI had more disadvantages of long-term complications such as rupture and capsular contracture^22^. In review articles by See^2^ and Yun^23^, immediate TEI showed relatively higher risk of complications compared to autologous reconstruction. In particular, capsular contracture was most observed complication in patients with PMRT after TEI. Regarding the RT fractionation, Kim et al. revealed that hypofractionation appeared to have comparable breast-related complications in patients with reconstruction compared with conventional fractionation^24,25^. Since complication itself could cause poor cosmetic outcomes^5^, results of these studies could be comparable with our findings. Thus, we found that AS detected by the f-AnoGAN deep learning mode could be feasible in evaluating the cosmetic outcomes.

For the analysis of longitudinal data, we established LME model to incorporate time variable. Since LME models are well suited for analysis of longitudinal data, we tested whether days after RT impacted on cosmetic outcomes considering other covariates. We identified that days after RT were associated with increased AS significantly, in contrast, age was not related with increased AS. This suggested the possibility of the chronic impact on cosmetic outcome induced by RT. Previous studies revealed that the timing of breast reconstruction, the reconstruction type, RT techniques (3D conformal vs. intensity-modulated RT), and RT fractionation could affect aesthetic satisfaction after breast reconstruction^2,5,22,26,27^. Also, there were several studies reporting that higher BMI might increase the complication after breast reconstruction^28,29^. However, reconstruction type, RT fractionation, either interaction with days, and BMI were not related with AS in current study. Instead, we found that patients with tissue expander demonstrated higher AS than those with autologous consistently across time after RT. Further difference between them has been widened with passage of time. This finding is consistent with the results of a systemic review and meta-analysis that autologous reconstruction yields better satisfactory breast and overall outcomes^30^.

There are several limitations. The current study is based on small dataset retrospectively collected from one institution, entailing an inherent bias for patient selections. For example, in terms of implant, only patients reconstructed with TEI were evaluated, not with permanent implant. Although mastectomy reconstruction with permanent implant is performed recently in our institution, those cases were excluded from the study population due to the short follow-up period less than 2 years. Long-term follow-up more than 5 years may be needed to consolidate the feasibility of AS. AS in patient who completed TEI followed by permanent implant was not evaluated in current study. Nevertheless, we used a relatively large number of normal data images, training the f-AnoGAN model that could differentiate anomaly from normal well. Since AS is generated as continuous value by the f-AnoGAN model, this score system might complement the categorical results from BCCT.core software. For benchmarking, the BCCT.core program cannot be used since it is not currently available on the official website. Also, because cosmetic evaluation using f-ANOGAN in our study is a completely novel method, it was difficult to find other deep learning models to compare with. Therefore, we plan to verify the clinical usefulness of the AS detected by f-ANOGAN through a large multicenter study.

Taken together, our findings might be helpful for physicians to evaluate cosmetic outcomes using regular follow-up CT images in patients who received mastectomy and immediate reconstruction. Importantly, AS should be validated in prospective study settings.

## Methods

This study is reviewed and approved by Seoul National University Bundang Hospital institutional review board (Approval number: B-2102–667–111). All procedures performed in this study involving human participants were in accordance with the ethical standards of the institutional and/or national research committee and with the 1964 Declaration of Helsinki and its later amendments or comparable ethical standards. Due to the retrospective study, the requirement for informed consent from participants was exempted.

### Subjects and data preprocessing

To train the GAN model with normal breast image data, altogether 251 breast cancer patients who underwent breast-conserving surgery and then who received post-operative RT were collected. As an evaluation dataset, we retrospectively reviewed 61 breast cancer patients who underwent mastectomy followed by immediate breast reconstruction with autologous tissue or tissue expander. In each patient, three time point of CT images were collected: CT simulation before RT (Pre-RT), 1 year after RT completion (Post-1Y), and 2 years after RT completion (Post-2Y). In order to preprocess data for using fast anomaly generative adversial network (f-AnoGAN), we reconstructed each patient’s CT Digital Imaging and Communications in Medicine (DICOM) images into 3-dimsional (3D) volume. Isosurface of 3D volume was generated with positioning of breast or chest wall as front-forward. Color of background and isosurface of volume was chosen to white. Then, image size was resized to 500 × 500 pixels. DICOM import, 3D reconstruction, isosurface acquisition, and image preprocessing were performed by using MATLAB 2021a ﻿(The MathWorks Inc, United States).

### Fast anomaly generative adversial network (f-AnoGAN) and calculation of anomaly score

We used the published the f-AnoGAN algorithm^18^ to develop the GAN-based model. The f-AnoGAN was intended for anomaly GAN to be performed fast. The network is composed of generative model, an encoder mapping new data to the latent space, and discriminator detecting anomalies. The f-AnoGAN is characterized to replace iterative mapping process with a learned mapping process from image to latent space, dramatically improving speed. Therefore, the f-AnoGAN technique exceedingly improved the process speed compared with other anoGAN algorithms.

We followed the methodology of the original study^18^, which is summarized here as follows:

We trained the generator network *G* and the discriminator network *D* to train the Wasserstein GAN, establishing a latent representation of normal breast images.

1 $$\mathop {{\min }}\limits_G \mathop {{\max }}\limits_D E_{X \sim Pr}\left[ {\log D\left( X \right)} \right] + E_{X\prime \sim Pg}\left[ {\log \left( {1 - D\left( {X^\prime } \right)} \right)} \right]$$

where $X^\prime$ is the generated instance of *G*(*z*) and the *z* is the learned latent feature. The *G*(*z*) is able to generate image $X^\prime$ from *z*: $z \to X^{\prime}$. However, the representation within the latent space for a given image is unknown. Thus, the encoder network (*E*) is required to map images to the latent space, *E*(*X*) = *X* → *z*. To find the best *z* corresponding to given image *X*, we trained an encoder based on izi_f_ architecture suggested in original study. The loss function of izi encoder training, izi_f_, is defined as follows:

2 $$Loss_{izi} = Loss_{images} + k \ast Loss_{features}$$

3 $$Loss_{images} = \frac{1}{n}\left\| {X - X{^{\prime}} } \right\|^2$$

4 $$Loss_{features} = \frac{1}{m}\left\| {f\left( X \right) - f\left( {X^\prime } \right)} \right\|^2,$$

where *k* is the weighting factor. *Loss*_*images*_ is the mean squared error (MSE) loss between real image *X* and the reconstructed image $X^{\prime}$, and *Loss*_*features*_ is the discriminator feature space loss based on the activation of the intermediate layer of *D* and *m* dimensionality of intermediate feature. Finally, AS was calculated by weighted sum of discriminator feature residual error and an image reconstruction error as follows:

Given an image X,

5 $${{{\mathrm{Anomaly}}}}\;{{{\mathrm{Score}}}} = \left( {1 - \lambda } \right) \ast {{{\mathrm{R}}}}\left( {\mathrm{X}} \right) + {\uplambda} \ast {{{\mathrm{D}}}}\left( {{{\mathrm{X}}}} \right)$$

where λ represents a weight coefficient, R(X) represents reconstruction loss between X and corresponding image in latent space, and D(X) represents dissimilarity features from discriminator. In current study, λ was determined to be 0.95.

We adopted publicly available implementation code from repository (https://github.com/A03ki/f-AnoGAN). Hyperparameters for training was determined as follows: Optimizer = Adam, learning rate = 0.001, batch size = 32, the number of epoch = 7000, dimension of latent space = 128, input image size = 256 by 256, β1 = 0.5, β2 = 0.999. The training and testing were performed with a GeForce GTX 1080Ti graphics processing unit (NVIDIA, Santa Clara, CA, USA). The higher AS indicates worse cosmetic outcomes.

The process of developing f-AnoGAN model and generation of AS is visualized in Fig. 1. Firstly, CT at the time of RT planning was acquired, reconstructed into 3D volume, and its frontal surface was captured as 2D image. Then, these 2D images were used to train f-AnoGAN model to differentiate other images and scoring the degree of anomaly. After training f-AnoGAN model, we applied this model for patients with reconstruction who received adjuvant RT. In a time-series manner, we acquired CT images from those patients: at Pre-RT, Post-1Y, and Post-2Y. AS was generated as standardized z-score based on the model trained by normal breast image data.

### Statistical analysis

To compare AS among groups (Pre-RT, Post-1Y, and Post-2Y), we performed paired t-test. Complication events included hematoma, wound infection, wound dehiscence, reconstructive flap necrosis, flap contracture, fat necrosis, capsular contracture, implant leakage/rupture/deflation, breast pain, and breast lymphedema. Among these, major complication was defined as any event which needs reoperation or rehospitalization. This definition is consistent with previous papers^24,25^. We compared AS among three time points (Pre-RT, Post-1Y, Post-2Y) stratifying major complication and type of reconstruction. For longitudinal analysis, a linear mixed-effect model (LME) was employed for analyzing time-effect on the change of AS. AS was response variable, and patient identifiers were random effect in the model. Model covariates included type of reconstruction, days after baseline, RT fractionation scheme, type of mastectomy, irradiation to supraclavicular node (SCN), irradiation to internal mammary nodes (IMN), boost RT, RT modality, the presence of major complication, age, and body mass index (BMI, kg/m^2^). These variables were selected as potential factors related with cosmetic results, based on previous studies^22,24,25,31^. Using LME model with those variables, we investigated longitudinally interactive relationships of type of reconstruction and RT fractionation scheme with respect to AS. All statistical tests were performed, and residual plots were depicted by using STATA/MP version 15.0 (StataCorp, College Station, TX). Bar graphs were generated by PRISM version 9.1.1.

### Reporting summary

Further information on research design is available in the Nature Research Reporting Summary linked to this article.

## Supplementary information

Reporting Summary

## Data Availability

Research data are stored in an institutional repository and will be shared upon request to the corresponding author.

## Code availability

Available code used for current analysis including model development and testing is available at publicly repository. (https://github.com/bigwiz83/SNUHRO_anoGAN_for_Cosmesis).
