# Author Correction: Feasibility of anomaly score detected with deep learning in irradiated breast cancer patients with reconstruction

**DOI:** 10.1038/s41746-022-00688-5

**Published:** 2022-09-07

**Authors:** Dong-Yun Kim, Soo Jin Lee, Eun-Kyu Kim, Eunyoung Kang, Chan Yeong Heo, Jae Hoon Jeong, Yujin Myung, In Ah Kim, Bum-Sup Jang

**Affiliations:** 1 Department of Radiation Oncology, Seoul National University Hospital, Seoul, Korea; 2 College of Medicine, Seoul National University, Seoul, Korea; 3 Department of Surgery, Seoul National University Bundang Hospital, Seoul National University College of Medicine, Seongnam, Korea; 4 Department of Plastic and Reconstructive Surgery, Seoul National University Bundang Hospital, Seongnam, Korea; 5 Department of Radiation Oncology, Seoul National University Bundang Hospital, Seongnam, Korea

**Keywords:** Outcomes research, Biotechnology

Correction to: *npj Digital Medicine* 10.1038/s41746-022-00671-0, published online 23 August 2022

In this article the author name Eun-Kyu Kim was incorrectly written as Eun Kyu Kim. The original article has been corrected.

